# Characteristics of Gut Microbiota in Children With Biliary Atresia After Liver Transplantation

**DOI:** 10.3389/fphys.2021.704313

**Published:** 2021-06-29

**Authors:** Wei Song, Li-Ying Sun, Zhi-Jun Zhu, Lin Wei, Wei Qu, Zhi-Gui Zeng, Yun-Sheng Yang

**Affiliations:** ^1^Liver Transplantation Center, National Clinical Research Center for Digestive Diseases, Beijing Friendship Hospital, Capital Medical University, Beijing, China; ^2^Clinical Center for Pediatric Liver Transplantation, Capital Medical University, Beijing, China; ^3^Department of Intensive Care Unit, Beijing Friendship Hospital, Capital Medical University, Beijing, China; ^4^Department of Gastroenterology and Hepatology, The First Medical Center, Chinese PLA General Hospital, Beijing, China

**Keywords:** biliary atresia, liver transplantation, gut microbiota, metagenomic sequencing, infection

## Abstract

**Background and Aims:**

Biliary atresia (BA) is an idiopathic neonatal cholestasis and is the most common indication in pediatric liver transplantation (LT). Previous studies have suggested that the gut microbiota (GM) in BA is disordered. However, the effect of LT on gut dysbiosis in patients with BA has not yet been elucidated.

**Methods:**

Patients with BA (*n* = 16) and healthy controls (*n* = 10) were recruited. In the early life of children with BA, Kasai surgery is a typical procedure for restoring bile flow. According to whether BA patients had previously undergone Kasai surgery, we divided the post-LT patients into the with-Kasai group (*n* = 8) and non-Kasai group (*n* = 8). Fecal samples were collected in both the BA and the control group; among BA patients, samples were obtained again 6 months after LT. A total of 40 fecal samples were collected, of which 16 were pre-LT, 14 were post-LT (8 were with-Kasai, 6 were non-Kasai), and 10 were from the control group. Metagenomic sequencing was performed to evaluate the GM.

**Results:**

The Kruskal-Wallis test showed a statistically significant difference in the number of genes between the pre-LT and the control group, the pre-LT and the post-LT group (*P* < 0.05), but no statistical difference between the post-LT and the control group. Principal coordinate analysis also showed that the microbiome structure was similar between the post-LT and control group (*P* > 0.05). Analysis of the GM composition showed a significant decrease in *Serratia*, *Enterobacter*, *Morganella*, *Skunalikevirus*, and *Phifllikevirus* while short chain fatty acid (SCFA)-producing bacteria such as *Roseburia*, *Blautia*, *Clostridium*, *Akkermansia*, and *Ruminococcus* were increased after LT (linear discriminant analysis > 2, *P* < 0.05). However, they still did not reach the normal control level. Concerning functional profiles, lipopolysaccharide metabolism, multidrug resistance, polyamine biosynthesis, GABA biosynthesis, and EHEC/EPEC pathogenicity signature were more enriched in the post-LT group compared with the control group. Prior Kasai surgery had a specific influence on the postoperative GM.

**Conclusion:**

LT partly improved the GM in patients with BA, which provided new insight into understanding the role of LT in BA.

## Introduction

The gut microbiome plays an important role in human health and comprises about 10^14^ resident microorganisms, including bacteria, viruses, fungi, and archaea. Among these, bacteria are dominant (Lozupone et al., 2012). Gut microbiota (GM) provides energy and nutrients to intestinal epithelial cells by producing short-chain fatty acids (SCFAs). It can also promote gut immunity by acting on the toll-like receptor (TLR) (Singh et al., 2017). Inversely, it will lead to systemic infections when the microbiota balance is broken (Zhang et al., 2015). Accumulating literature suggests that GM imbalance occurs in many diseases such as liver disease (Miura and Ohnishi, 2014; Tilg et al., 2016), metabolic disease (Burcelin, 2017), cardiovascular disease (Ahmadmehrabi and Tang, 2017; Kazemian et al., 2020), inflammatory bowel disease (Nishida et al., 2018) and neurodegenerative disease (Quigley, 2017), and it plays a driving role in disease progression. By studying the role of GM in the management of illness, therapeutic measures have been developed to intervene in disease progression or complications, such as antibiotics, probiotics, prebiotics, and symbiotics.

Concerning end-stage liver disease, liver transplantation (LT) is the only cure. Biliary atresia (BA) is one of the most common indications for LT in children. BA is a devastating fibro-inflammatory disease that affects both extra- and intrahepatic bile ducts, its incidence ranges from 1/5,000 to 1/18,000. Prolonged neonatal jaundice, pale stools, and conjugated hyperbilirubinemia are the typical symptoms. Moreover, BA is a major cause of pediatric cholestasis (Feldman and Mack, 2015). Kasai surgery is typically performed to restore bile flow in early BA; however, up to 80% of patients will eventually require LT. Thus, Kasai is the standard first procedure for BA, and LT is reserved as a complementary and final therapy. Sun et al. (2017) demonstrated that LT significantly improved GM in adults with liver disease; GM disorders were also demonstrated in children with BA in our previous study. Nevertheless, the effect of LT on BA has not been described. Wang et al. (2016) summarized the impact of prior Kasai surgery on the clinical outcomes of LT and concluded that it might increase the risk of postoperative infection. However, the effect of prior Kasai surgery on the GM after LT is unknown.

Therefore, the present study aimed to investigate the effect of LT and describe the status of GM after LT in children with BA and the effect of prior Kasai surgery on the postoperative GM. Metagenomic sequencing was performed to evaluate the composition and function of the gut microbiome in feces.

## Materials and Methods

### Study Design

We collected stool samples from BA and healthy subjects at Beijing Friendship Hospital, Capital Medical University. Samples from BA patients were collected before and 6 months after LT. The point of post-LT timeframe was chosen to avoid immediate and transient postoperative complications (Jiang et al., 2018). Patients and controls were age-matched. Their dietary habits were similar. The following conditions in the enrolled group were met: (1) No digestive diseases within 1 month; (2) No antibiotics and probiotics within 1 month; and (3) age < 3 years. The diagnosis of BA was confirmed by intraoperative cholangiography and liver biopsy. The liver function index of controls was in the normal range. In patients with BA, preoperative data included age, body mass index (BMI), sex, pediatric end-stage liver disease (PELD) score, liver enzymes (ALT: alanine aminotransferase, AST: aspartate aminotransferase, ALP: alkaline phosphatase, GGT: γ-glutamyltransferase), and inflammatory cytokines (IL-2: interleukin-2, IL-4: interleukin-4, IL-6: interleukin-6, IL-10: interleukin, TNFα: tumor necrosis factor α, IFN-γ: interferon-γ), We examined liver function indexes and inflammatory factors again after LT. Metagenomic sequencing can analyze species composition and functional metabolism in greater depth.

### Sample Collection and DNA Extraction

All samples were stored at –80°C within four hours of collection. Bacterial DNA was extracted using the QIAamp Fast DNA Stool Mini Kit (51604; Qiagen, Hilden, Germany). Ten micrograms of stool sample was weighed in a centrifuge tube, approximately 25 mg of pre-cooled submerged beads were added, and then 200 μL of acetonitrile/methanol (v/v = 8:2) solvent containing 10 μL of an internal standard for homogenous mixing was added and centrifuged at 13,500 rpm and 4°C for 20 min to remove proteins. After centrifugation, 10 μL supernatant was obtained, diluted with 90 μL of 1:1 acetonitrile/methanol (v/v = 80/20) and ultrapure water mixed solvent, shaken and centrifuged for analysis. The injection volume was 5 μL. The detailed extraction steps followed the QIAamp^®^ Fast DNA Stool Mini Handbook instructions. The DNA concentration was measured with a NanoDrop (Thermo Fisher Scientific, MA, United States) and Qubit^®^2.0 (Invitrogen, Carlsbad, CA, United States), and the molecular size was estimated by agarose gel electrophoresis.

### Metagenomic Sequencing

Following the Illumina TruSeq DNA Sample Prep v2 Guide (San Diego, CA, United States), we constructed the DNA paired-end libraries with an insert size of 500 bp for the 40 stool samples (16 from pre-LT, 14 from post-LT, and 10 from controls). The quality of all libraries was evaluated using an Agilent 2100 bioAnalyzer (Agilent Technologies, Wokingham, United Kingdom) and an Agilent 2100 DNA 1000 kit. All samples were subjected to 150-bp paired-end sequencing on an Hiseq X-ten platform (Illumina). Illumina raw reads were screened according to the following criteria: (1) adaptor contamination reads were removed; (2) reads containing more than three ambiguous N bases were removed; (3) reads containing low quality (Q < 20) bases were trimmed; (4) reads containing less than 60% of high-quality bases (Phred score ≥ 20) were deleted.

Clean reads were subjected to bacterial genomes from the National Center for Biotechnology Information GenBank with SOAPaligner (version 2.21), and reads mapped to the host genome were abandoned.

For species classification, the NCBI database^1^ (National Center for Biological Information) was used to align the clean reads with known bacteria, fungi, viruses, and archaea by SOAPaligner 2.21. With regard to the functional profiles, the non-redundant genes were annotated against the KEGG (Kyoto Encyclopedia of Genes and Genomes) database using BLAST (v. 2.2.28 +). When the assembled protein sequence was similar (score ≥ 60 and *E* < 1e–5) to a protein sequence in the database, the assembled protein was considered to play the same role as the database protein. The relative abundance of all orthologous genes was accumulated to generate the relative abundance of each KO (KEGG ortholog).

### Statistical Analysis

Statistical analyses were completed using R v.3.3.1. The non-parametric Wilcoxon test and Kruskal-Wallis test were performed to analyze the different species or functional profiles. Differences were considered significant at *P* < 0.05 or a false discovery rate (FDR) < 0.1. Differences in taxa and functional profiles at various levels were evaluated by Wilcoxon rank testing and linear discriminant analysis (LDA) effect size (LEfSe), where a higher LDA score reflected a more prominent difference in abundance between the different groups. The LDA score cut-off of 2.0 indicated a significant difference. Spearman’s rank correlation was used to examine associations among variables. Fishtaco was used to analyze the contribution of species to differential metabolic pathways.

## Results

### Study Population

Forty stool samples were obtained from twenty-six participants enrolled in this study, including 16 pre-LT, 14 post-LT, and 10 control samples. In the post-LT group, two patients had diarrhea at the sampling time, which did not meet the inclusion criteria of this study, so their samples were not collected. All BA patients received orthotopic liver transplantation (OLT). After the operation, they were given immunosuppressive therapy with tacrolimus and methylprednisolone. In the first 5–7 days after LT, the patients received broad-spectrum antibiotics with cephalosporin to prevent infection. The clinical data of the enrolled individuals and demographic information are shown in Tables 1, 2. The results of metagenomic sequencing and assembly data in each sample are listed in Supplementary Table 2.

**TABLE 1:** Demographic information and clinical data on patients with BA (pre-LT) and control individuals.

	BA (*n* = 16)	Control (*n* = 10)	*P*-value
Age, months, median (min, max)	7.8(4.8,21)	8(5,20)	>0.05
**Gender**			>0.05
Female, n (%)	10(62.5%)	6(60%)	
Male, n (%)	6(37.5%)	4(40%)	
BMI, kg/m^2^, median (min, max)	16.94(11.26,21.33)	18.79(15.85,20.09)	> 0.05
**Hepatic function, median (min, max)**			
ALT, U/L	151.00(21.00,439.00)	14.85(10.20,20.10)	< 0.05
AST, U/L	211.90(45.40,724.30)	39.35(27.20,43.90)	< 0.05
ALP, U/L	610.5(296.00,1409.00)	236.20(192.60,317.00)	< 0.05
GGT, U/L	180.50(26.00,980.00)	11.30(9.00,16.30)	< 0.05
TBA, μmol/L	163.8(57.00,356.00)	1.30(0.30,4.70)	< 0.05
TBIL, μmol/L	278.50(9.02,898.13)	5.70(2.50,11.60)	< 0.05
**Inflammatory cytokines (min, max)**			
IL-2, pg/mL	73.63(0.96,262.02)	3.58(0.96,5.32)	< 0.05
IL-4, pg/mL	39.47(3.60,143.85)	2.22(1.77,11.09)	< 0.05
IL-6, pg/mL	70.87(7.38,233.53)	2.98(1.23,14.92)	< 0.05
IL-10, pg/mL	25.66(2.56,61.84)	0.86(0.27,4.28)	< 0.05
TNFα, pg/mL	31.55(2.20,117.86)	2.09(1.11,3.26)	< 0.05
IFN-r, pg/mL	85.38(2.10,341.05)	2.10(2.09,2.11)	< 0.05
PELD	19(1,39)	Not Applicable	–

**BA, biliary atresia; LT, liver transplantation; ALT, alanine aminotransferase; AST, aspartate aminotransferase; ALP, alkaline phosphatase; GGT, γ-glutamyltransferase; TBA, total bile acids; TBIL, total bilirubin; IL-2, interleukin-2; IL-4, interleukin-4; IL-6, interleukin-6; IL-10, interleukin-10; TNFα, tumor necrosis factor α; IFN-γ, interferon-γ; PELD, Pediatric End-stage Liver Disease.**

**TABLE 2:** Characteristics of patients in the post-LT setting at the time of sample collection.

Variable	Value
**Hepatic function, median (min, max)**	
ALT, U/L	20.50(11.00,57.00)
AST, U/L	35.60(23.00,109.60)
ALP, U/L	266.50(129.00,510.00)
GGT, U/L	17.00(10.00,137.00)
TBA, μmol/L	9.24(1.50,20.30)
TBIL, μmol/L	8.45(3.84,18.54)
**Inflammatory cytokines (min, max)**	
IL-2, pg/mL	55.07(17.84,94.89)
IL-4, pg/mL	18.38(6.02,37.33)
IL-6, pg/mL	32.15(7.93,50.39)
IL-10, pg/mL	2.96(1.09,6.05)
TNFα, pg/mL	12.65(6.85,26.94)
IFN-r, pg/mL	15.84(2.10,72.22)
Sampling time (Since the last antibiotic use), days, median (min, max)	136(125,140)
**Postoperative complications**	
Infection	3
Portal thrombosis	1

**BA, biliary atresia; LT, liver transplantation; ALT, alanine aminotransferase; AST, aspartate aminotransferase; ALP, alkaline phosphatase; GGT, γ-glutamyltransferase; TBA, total bile acids; TBIL, total bilirubin; IL-2, interleukin-2; IL-4, interleukin-4; IL-6, interleukin-6; IL-10, interleukin-10; TNFα, tumor necrosis factor α; IFN-γ, interferon-γ.**

### Impact of LT on GM in BA Individuals

**FIGURE 1:**
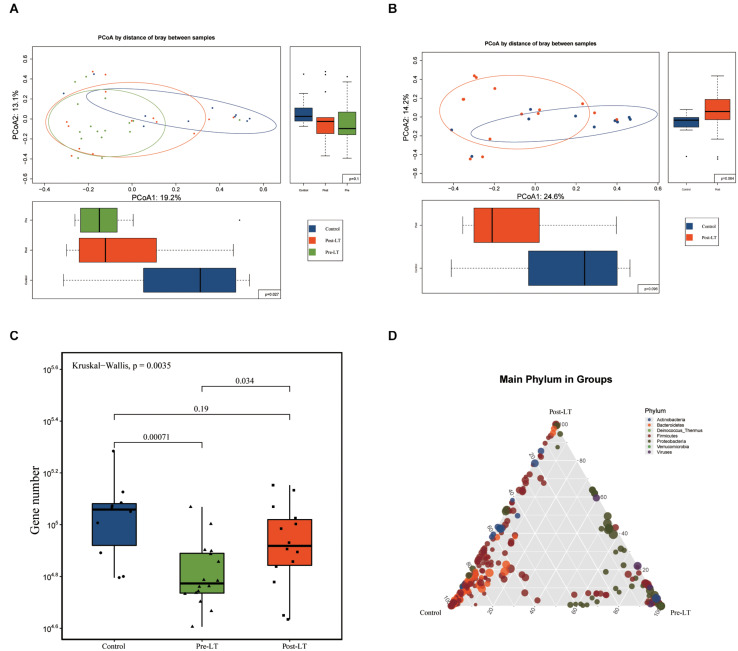
**(A)** Structural changes in the gut microbiota in pre-LT, post-LT, and control individuals (principal coordinate analysis based on the Bray–Curtis distance of species abundance). **(B)** Structural changes in the gut microbiota in pre-LT and post-LT individuals (principal coordinate analysis based on the Bray–Curtis distance of species abundance). **(C)** Different gene numbers between the three groups (Wilcoxon rank-sum test). **(D)** Ternary plot of different species. The three vertices represent the three different groups, the points represent the different species at the species level, and the size of the points represent the average abundance in the three groups of samples. Points that are enriched in a certain color at a vertex indicate higher species abundance at the phylum level represented by the color in the grouping.

Principal coordinate analysis (PCoA) was performed to analyze GM composition structure between the three groups (pre-LT, post-LT, and control group), which showed that the structure of the post-LT group was between the pre-LT group and the control group (Figure 1A). Figure 1B shows that the microbiome structure was similar between the post-LT and control group (*P* > 0.05). The Kruskal-Wallis test showed a statistically significant difference in the number of genes between the pre-LT and the control group, the pre-LT, and the post-LT group, but no statistical difference between the post-LT and the control group (Figure 1C). The ternary plot also indicated the GM composition differences between the three groups (Figure 1D). More species were seen in the control group than in the other two groups. Bacteroidetes and Firmicutes were enriched in the control group. Proteobacteria was predominant in the pre-LT group, and the post-LT group consisted of a mixture of control and pre-LT species. Taken together, LT significantly changed GM composition in patients with BA.

**FIGURE 2:**
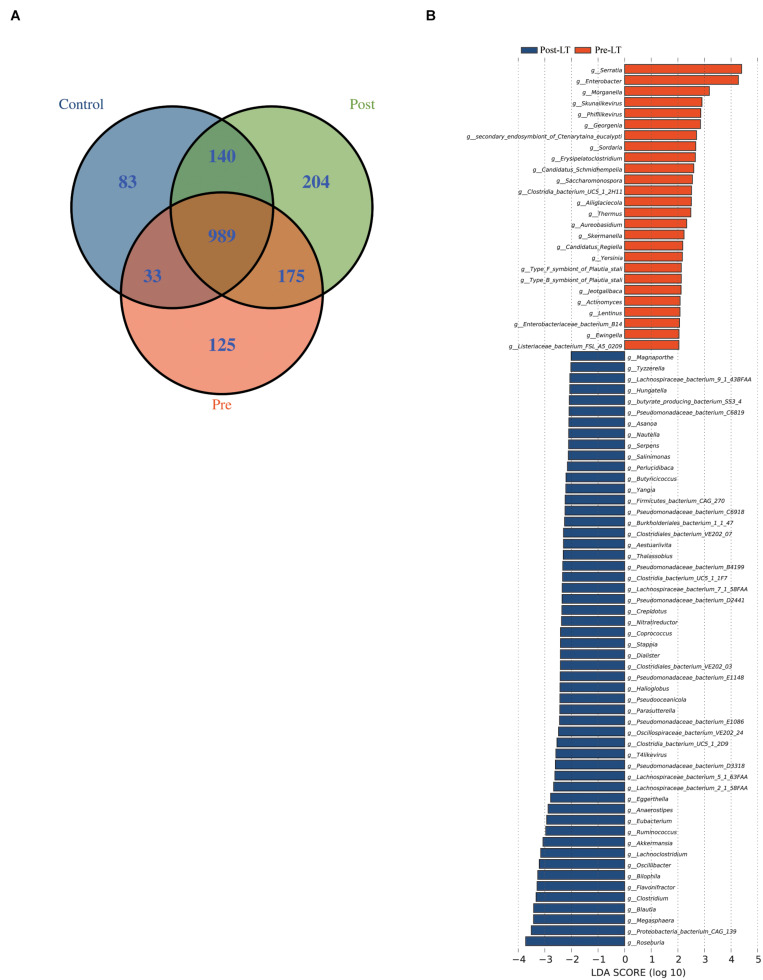
**(A)** Venn diagram of the pre-LT, post-LT, and control groups. **(B)** LEfSe analysis of the gut microbiota composition between the pre-LT and post-LT groups. Histogram of the LDA scores calculated for the differential abundance of genera in the two groups. Different colors represent different groups.

The Venn diagram demonstrates the shared and unique genera between the three groups (Figure 2A). Nine hundred and eighty-nine genera were shared among the three groups, while 83, 125, and 204 were unique in the control, pre-LT, and post-LT groups, respectively. Moreover, 1,129 genera were shared between the control and post-LT groups, and 1,022 genera were shared between the control and pre-LT groups. It also proved a shift in GM composition to the control group after LT surgery.

**FIGURE 3:**
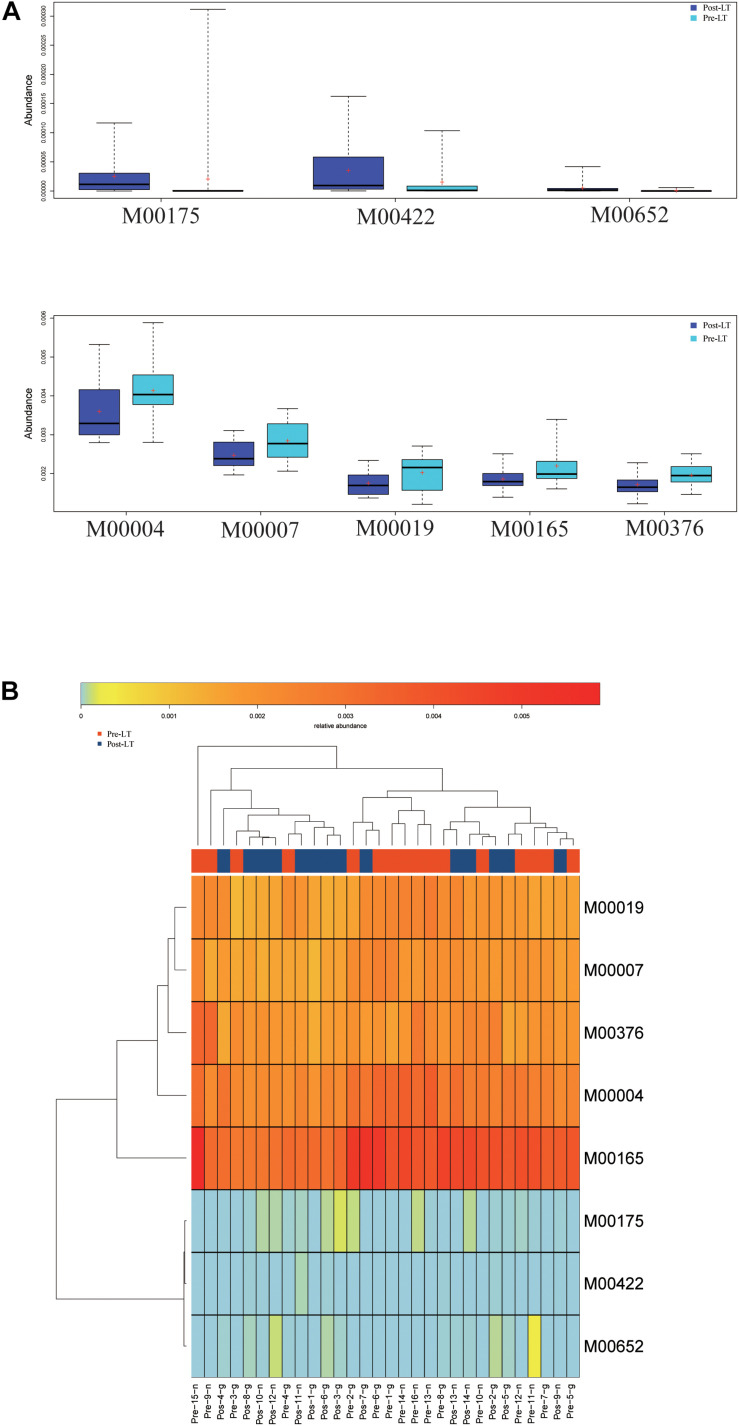
**(A)** Functional profiles with significantly different abundances at the module level between the pre-LT and post-LT groups (Wilcoxon rank-sum test, *P* < 0.05). Different colors represent different groups. **(B)** Hierarchical clustering between differential modules and samples.

**FIGURE 4:**
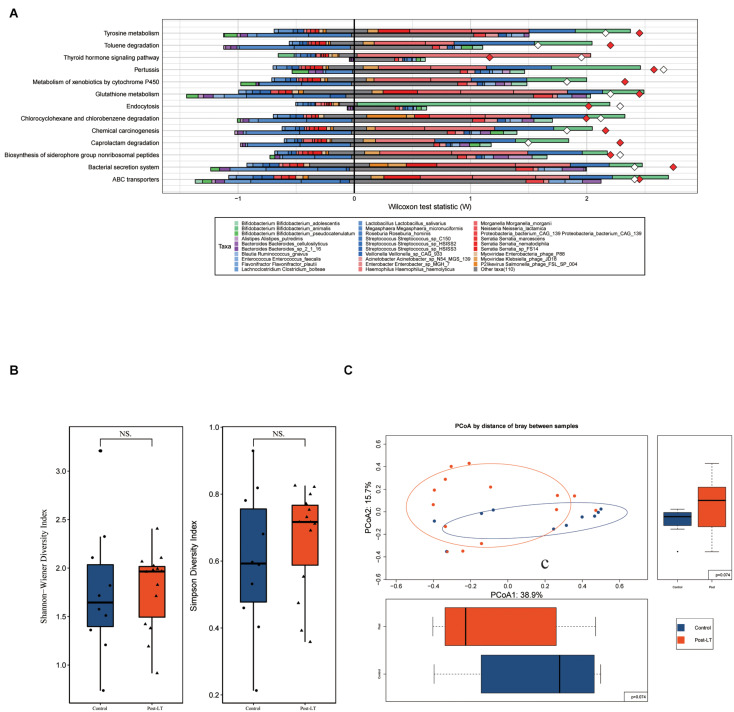
**(A)** Fishtaco analysis of species contribution to metabolic pathways between the pre-LT and post-LT groups. The *x*-axis represents the Wilcoxon test statistic scores, and the *y*-axis the related functions. The driving factors for each differential function transformation were divided into four parts, represented by a histogram in two directions. Gut microbiota in the pre-LT group that drove the increase in the corresponding functional abundance (top right); Gut microbiota in the pre-LT group that inhibited the increase in the corresponding functional abundance (top left); Gut microbiota in the post-LT group that drove the increase in the corresponding functional abundance (bottom right); and gut microbiota in the post-LT group that inhibited the increase in the corresponding functional abundance (bottom left). Different color bars represent the corresponding species. The longer the bar, the greater the driving or inhibiting effect of the species on the corresponding function. **(B)** Alpha diversity between the post-LT and control groups (box plot of richness, Shannon–Wiener Index, and Simpson Index). **(C)** Beta diversity between the post-LT and control groups (principal coordinate analysis based on the Bray–Curtis distance of species abundance).

Next, LEfSe was performed to analyze the different species between the pre-LT and post-LT groups. It was shown that 26 and 54 genera were enriched in the pre-LT and post-LT groups, respectively. *Serratia*, *Enterobacter*, *Morganella*, *Skunalikevirus*, and *Phifllikevirus* were enriched in the pre-LT group, while *Roseburia*, *Blautia*, *Clostridium*, *Akkermansia*, *Ruminococcus*, and *Eubacterium* were enriched in the post-LT group (Figure 2B and Supplementary Table 3). Concerning functional profiles, the Wilcoxon test showed that M00004 (pentose phosphate pathway), M00007 (pentose phosphate pathway), M00019 (valine/isoleucine biosynthesis), M00165 (reductive pentose phosphate cycle), and M00376 (3-hydroxypropionate bi-cycle) were enriched in the pre-LT group. In contrast M00175 (nitrogen fixation), M00422 (acetyl-CoA pathway), and M00652 (vancomycin resistance) were enriched in the post-LT group (Figure 3A and Supplementary Table 4). An abundance clustering heat map of different modules shown in Figure 3B. In the pathways, lipid metabolism, amino acid metabolism, carbohydrate metabolism, energy metabolism, xenobiotics biodegradation and metabolism, and cell motility significantly increased after LT (Supplementary Figure 1). The contribution of GM to metabolic pathways indicated that *Serratia* spp., *Proteobacteria* bacterium CAG 139, *Enterobacter*, *Veillonella* sp. CAG 933, and *Streptococcus* spp. constituted in the pre-LT group (Figure 4A). These are all inflammatory pathogenic bacteria. The composition of GM and functional metabolism both improved after LT.

### Difference in GM Between the Post-LT and Control Groups

Alpha diversity was represented by the Shannon–Wiener Index and Simpson Index, and beta diversity by PCoA; there was no significant difference between the two groups (Figures 4B,C).

**FIGURE 5:**
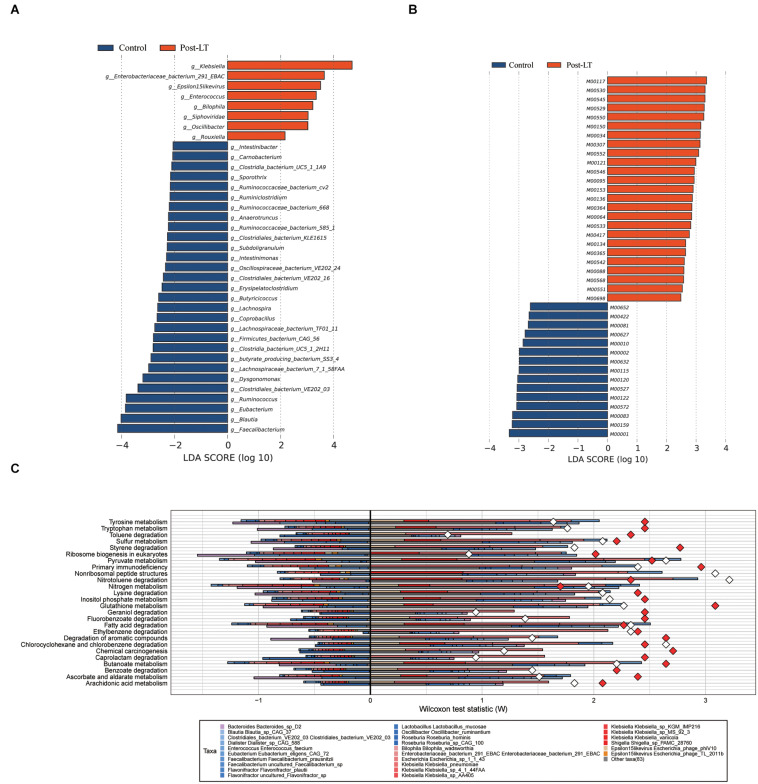
**(A)** LEfSe analysis of gut microbiota composition between post-LT and control groups. **(B)** LEfSe analysis of functional profiles between the two groups. Histogram of the LDA scores calculated for a differential abundance of genera in the two groups. Different colors represent different groups. **(C)** Fishtaco analysis of species contribution to metabolic pathways between the post-LT and control groups. The *x*-axis represents the Wilcoxon test statistic scores, and the *y*-axis the related functions. The driving factors for each differential function transformation were divided into four parts, represented by a histogram in two directions. Gut microbiota in the post-LT group that drove the increase in the corresponding functional abundance (top right); Gut microbiota in the post-LT group that inhibited the increase in the corresponding functional abundance (top left); Gut microbiota in the control group that drove the increase in the corresponding functional abundance (bottom right); and gut microbiota in the control group that inhibited the increase in the corresponding functional abundance (bottom left). Different color bars represent the corresponding species. The longer the bar, the greater the driving or inhibiting effect of the species on the corresponding function.

LEfSe analysis was used to further identify different species between the two groups. At the genus level, there were 37 different species. *Enterobacteriaceae* bacterium 291 EBAC, *Oscillibacter*, *Klebsiella*, *Enterococcus*, *Epsilon15likevirus*, *Rouxiella*, *Bilophila*, and *Siphoviridae* were enriched in the post-LT group. In contrast, *Faecalibacterium*, *Blautia*, *Eubacterium*, *Clostridiales* bacterium VE202 03, and *Ruminococcus* were enriched in the control group (Figure 5A and Supplementary Table 5). There were 41 and 68 different species enriched in the post-LT and control group at the species level, respectively. *Shigella* sp. PAMC 28760, *Klebsiella* sp. KTE92, *Enterobacteriaceae* bacterium 291 EBAC, *Klebsiella pneumoniae*, Escherichia phage phiV10, *Klebsiella* sp. MS 92 3, *Klebsiella variicola*, *Klebsiella* sp. D5A, *Klebsiella* sp. KGM IMP216, *Klebsiella* sp. 4 1 44FAA, and Escherichia phage TL 2011b were enriched in the post-LT group, while *Faecalibacterium prausnitzii*, *uncultured Faecalibacterium* sp., *Roseburia* sp. CAG 100, *Bacteroides* sp. D2, butyrate-producing bacterium SS3 4, *Clostridiales* bacterium VE202 03, *Ruminococcus lactaris*, *Eubacterium eligens*, *Eubacterium eligens* CAG 72, and *Blautia* sp. CAG 37 were enriched in the control group (Supplementary Figure 2 and Supplementary Table 6).

In functional metabolism, ABC transporters and Glycolysis/Gluconeogenesis were abundant in the post-LT group (Supplementary Table 7). LEfSe analysis showed that pathways related to lipopolysaccharide metabolism (M00064), multidrug resistance (M00698), cysteine and methionine metabolism (M00034), polyamine biosynthesis (M00134), GABA biosynthesis (M00136), and EHEC/EPEC pathogenicity signature (M00542) were enriched in the post-LT group. In contrast, beta-lactam resistance (M00627), vancomycin resistance (M00652), methane metabolism (M00422), and lysine metabolism (M00527) were enriched in the control group (Figure 5B, and Supplementary Table 8). Fishtaco analysis indicated that the composition of the GM that drove the metabolic pathways was different between the two groups (Figure 5C). *Epsilon15likevirus, Shigella* sp. PAMC 28760, *Klebsiella* spp., *Escherichia* sp. 1 1 43, *Enterobacteriaceae* bacterium 291 EBAC, and *Bilophila wadsworthia* played major driving roles in the post-LT group. In contrast, *Roseburia* sp. CAG 100, *Roseburia hominis*, *Oscillibacter ruminantium*, *Lactobacillus mucosa*e, *Flavonifractor* spp., and *Faecalibacterium prausnitzii* were the primary driving species in the control group. Thus, the significantly different species between the post-LT and control groups were investigated; however, the overall structure of the GM was similar. In addition, the functional pathway was different between the two groups.

### Relationship Between GM and Clinical Indicators

**FIGURE 6:**
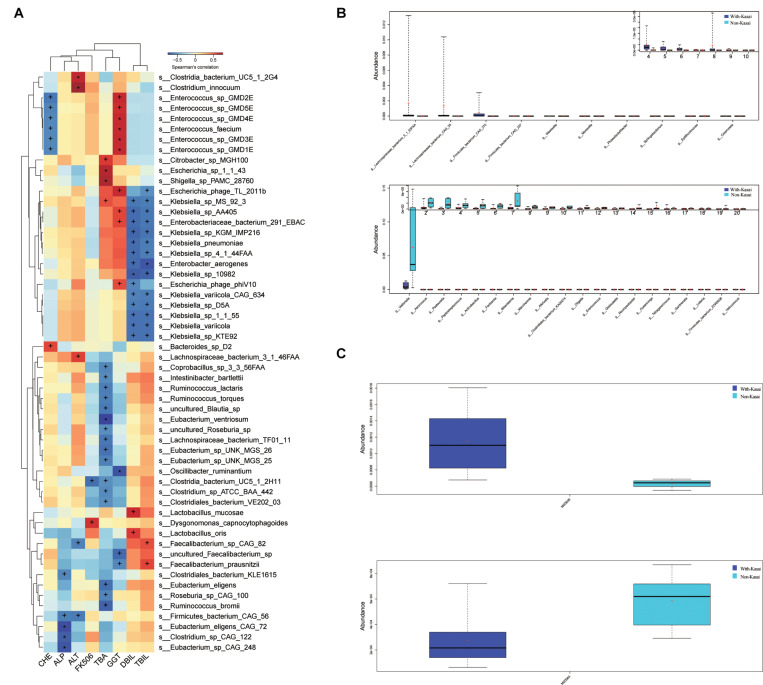
**(A)** Spearman correlations between species and clinical indices. The *x*-axis represents the environmental factors, and the *y*-axis the species. The depth of the color indicates the correlation between species and environmental factors (^+^*P* < 0.05, *P < 0.01). Blue denotes a negative correlation and red a positive correlation. **(B,C)** The effect of prior Kasai surgery on the gut microbiota after liver transplantation. **(B)** Species with significantly different abundances at the genus level in the with-Kasai and non-Kasai individuals (Wilcoxon rank-sum test, *P* < 0.05). The upper panel shows the abundance ranking of species in the with-Kasai group, and the lower panel the abundance ranking of species in the non-Kasai group. The *x*-axis represents the different taxa, and the *y*-axis the abundance of taxa. Different colors represent different groups. Lines inside the boxes indicate medians, “ + ” indicated means. **(C)** Functional profiles with significantly different abundances at the module level between the two groups (Wilcoxon rank-sum test, *P* < 0.05). Different colors represent different groups.

The relationship between different species identified in the post-LT and control groups and clinical indicators were analyzed by Spearman rank correlation (Figure 6A and Supplementary Table 9). It showed that *Erysipelatoclostridium* was positively correlated with ALT (*P* = 0.004, *r* = 0.71); *Eubacterium* sp. CAG 248 was negatively correlated with ALP (*P* = 0.004, *r* = −0.72); uncultured Faecalibacterium sp. was negatively correlated with GGT (*P* = 0.01, *r* = −0.65), while *Enterococcus* sp. GMD5E, *Enterococcus* sp. GMD3E, *Enterococcus faecium*, Escherichia phage TL 2011b, *Enterococcus* sp. GMD4E, and *Enterococcus* sp. GMD2E were all positively correlated with GGT (*P* = 0.01, *r* = 0.64; *P* = 0.01, *r* = 0.66; *P* = 0.01, *r* = 0.66; *P* = 0.01, *r* = 0.65; *P* = 0.01, *r* = 0.66; *P* = 0.01, *r* = 0.66, respectively); *Shigella* sp. PAMC 28760, *Citrobacter* sp. MGH100, and *Escherichia* sp. 1 1 43 were positively correlated with TBA (*P* = 0.004, *r* = 0.73; *P* = 0.02, *r* = 0.61; *P* = 0.006, *r* = 0.71, respectively), while uncultured Blautia sp., *Lachnospiraceae* bacterium TF01 11, *Eubacterium* sp. UNK MGS 26, *Ruminococcus bromii*, *Eubacterium ventriosum*, and *Eubacterium eligens* were negatively correlated with TBA (*P* = 0.02, *r* = −0.6; *P* = 0.02, *r* = −0.61; *P* = 0.02, *r* = −0.63; *P* = 0.01, *r* = −0.67; *P* = 0.002, *r* = −0.76; *P* = 0.02, *r* = −0.65, respectively). Taken together, liver functional indicators were positively correlated with species enriched in the post-LT group, while negatively correlated with the control group.

### Effects of Kasai Procedure on Postoperative GM

According to whether patients had previously undergone the Kasai procedure, we categorized them into the with- and non-Kasai groups. In total, 8 and 6 children were categorized in the with-Kasai and non-Kasai groups. The results showed that GM overall structure and diversity were similar between the two groups. With regard to GM composition, the abundance of several species was different, *Lachnospiraceae* bacterium 5 1 63FAA, *Lachnospiraceae* bacterium CAG 25, *Firmicutes* bacterium CAG 270, *Firmicutes* bacterium CAG 227, *Weeksella*, *Moraxella*, and *Phaeodactylibacter* exhibited higher abundance in the with-Kasai group, while *Veillonella*, *Aerococcus*, and *Actinobacillus* did not (Figure 6B and Supplementary Table 10). In the functional profiles, cysteine and methionine metabolism (M00609) was enriched in the with-Kasai group, while lipopolysaccharide metabolism (M00064) was enriched in the non-Kasai group (Figure 6C and Supplementary Table 11). Taken together, these findings showed that a prior Kasai procedure had a certain effect on the GM after LT.

## Discussion

BA is the major cause of neonatal cholestasis, and LT is currently the standard treatment (Sun et al., 2013; Feldman and Mack, 2015; Kasahara et al., 2017). Previous studies have suggested that the GM was altered during LT in adults with liver disease and animal models (Wu et al., 2012; Bajaj et al., 2017; Sun et al., 2017; Tian et al., 2018). In addition, the characteristics of GM after LT in children with BA have not been reported. The present study aimed to analyze the characteristics of GM in children with BA during LT.

Compared with the GM in patients before LT, the abundance of SCFA-producing bacteria (e.g., *Roseburia*, *Blautia*, *Clostridium*, *Akkermansia*, *Ruminococcus*, and *Eubacterium*) increased after LT. However, it was still lower than that in the control group. It demonstrated that LT partially improved dysbacteriosis in children with BA. With regard to functional profiles, it found that *Serratia spp.*, *Proteobacteria* bacterium CAG 139, *Veillonella* sp. CAG 933, and *Streptococcus* spp. were predominant in pathogenic related metabolic pathways in the pre-LT group. After LT, the driving effect of these bacteria on metabolic pathways was reduced. Besides, lipid metabolism, amino acid metabolism, carbohydrate metabolism, energy metabolism, xenobiotics biodegradation and metabolism, and cell motility increased in the post-LT group. Therefore, functional metabolism also improved after LT. Among the contributing microorganisms, *Streptococcus* spp. and *Veillonella* spp. have been reported to be enriched in the intestinal tract and biliary tract in immune liver diseases (Wei et al., 2019; Abe et al., 2020; Liwinski et al., 2020), which are associated with the etiology of autoimmune liver disease and damage to the epithelial barrier (Kugathasan et al., 2017); thus, LT can reduce the occurrence of epithelial inflammation from the perspective of the gut flora. On the other hand, selecting appropriate antibiotics for these bacteria for clinical treatment is useful for the intestinal epithelial barrier’s integrity and resisting inflammation.

Bile acids are critically important for maintaining a healthy gut microbiota. It interacts closely with the intestinal microbiota through the gut-liver axis. After LT, bile drainage into the intestine can reduce the pH of the intestinal environment, inhibit the growth of pathogenic bacteria, maintain the balance of intestinal microorganisms, and affect the composition of GM. Consistent with this, we observed that there was no statistical difference in either α or β diversity between the post-LT and control groups, which indicated that the composition of GM between the two groups was similar. Besides, to investigate whether the improvement in GM post-transplant is related to replacing the diseased liver or simply due to the establishment of bile flow to the intestine, we also compared the GM between the patients who gained successful bile flow (successful Kasai) and others were not (failed Kasai). We found that the abundances of 30 and 192 species were significantly increased after Kasai and LT (*P* < 0.05, Supplementary Table 13), respectively. Of the species that increased after Kasai, five species also increased significantly after LT (*Acidaminococcus*, *Catabacter*, *Clostridiales* bacterium VE202 03, *Erysipelatoclostridium*, and *Flavonifractor*), and 21 species increased but did not reach statistical significance. These results suggested that the change of GM after LT was greater than that after Kasai. Moreover, most of the changes in GM after Kasai also occurred after LT. We believed that the changes in GM after LT were not caused by bile acid drainage alone. In the post-LT group, Proteobacteria was the dominant phylum, which contains many known human pathogens, such as Enterobacteriaceae, which are thought to be markers of inflammation, epithelial dysfunction, and disease (Litvak et al., 2017; Liwinski et al., 2020). In addition, *Klebsiella*, *Enterobacteriaceae* bacterium 291 EBAC, *Enterococcus*, and *Bilophila* were enriched in the post-LT group. *Bilophila* is a sulfate-reducing bacterium whose abundance leads to excessive hydrogen sulfide production in the gut, stimulating an inflammatory response (Ye et al., 2018). *Enterococcus* and *Klebsiella* have been detected in various liver diseases (Ray, 2017; Fukui, 2019; Liu et al., 2019; Nakamoto et al., 2019) and were associated with a predisposition to penetrating complications in pediatric patients (Steck et al., 2011). *Enterobacteriaceae* bacterium 291 EBAC is a member of Enterobacteriaceae, also associated with epithelial inflammation and functional disorders. The number of SCFA-producing bacteria with anti-inflammatory effects was lower than that in the control group (Riviere et al., 2016). In early period of post-LT, recipients are at increased risk for developing infections. The use of antibiotic can deplete gut commensal bacteria, resulting in intestinal dysbiosis. Besides, immunosuppressive drugs may have caused the difference between the post-LT and control groups, and may affect the postoperative recovery of patients and increase the probability of complications. Additionally, Jiang et al. (2018) indicated that an optimal dosage of FK506 induced immunosuppression, normal graft function and stable gut microbiota following LT in rats. Therefore, regular detection of FK506 concentration is of positive significance for the homeostasis of intestinal flora. In addition, taking appropriate antibiotics or butyric acid may positively affect prognosis.

With regard to the functional profiles, ABC transporters and Glycolysis/Gluconeogenesis were abundant in the post-LT group. ABC transporters are an important cell-protective mechanism, which remove toxins and drugs from cells, and their enhanced metabolism may indicate intensive antibacterial ability in the intestine (Wang et al., 2019a; Ahmad et al., 2020). The metabolism of Glycolysis/Gluconeogenesis needs an oxygen-free environment, and its augmentation suggests that the anaerobic metabolism of sugar was active after LT (Wang et al., 2019b). Pathogen-related metabolism such as lipopolysaccharide metabolism and EHEC/EPEC pathogenicity signature were also abundant in the post-LT group. Lipopolysaccharide, also known as endotoxin, is the product of gram-negative bacteria, which can bind to TLR4 to initiate the inflammatory response (Cochet and Peri, 2017; Del Chierico et al., 2018). These results were in line with the bacterial composition in the post-LT group. Taken together, we believed that the changed functional profiles after LT are related to the different intestinal enrichment. Besides, GM are likely to affect the gut environment of hosts through itself composition and corresponding metabolism patterns and influence the development of disease.

Kasai operation could effectively delay the requirement of LT. Moreover, Li et al. (2019) had demonstrated that there was no significant difference in post-LT survival rate between the patients who had a prior Kasai and not. However, Urahashi et al. (2013) investigated that repeat Kasai might have a negative effect on patients who undergo LT for BA patients with potential lethal complications such as bowel perforation. Neto et al. (2015) also believed that a previous Kasai can increase post-LT complications as biliary complications and bowel perforations. Taken together, several studies have suggested that patients with a previous Kasai are at increased risk for bowel perforations, postoperative infection, and biliary complications from LT. Infection is a fatal complication after LT. Enterogenic infection represents a major complication in LT recipients (Zheyu and Lunan, 2006; Yuan et al., 2016; Mu et al., 2019); Therefore, we divided the post-LT group into two groups based on whether they had previously undergone the Kasai procedure to investigate the effect of Kasai surgery on the GM after LT. The results showed that some species and functional pathways were different between the two groups; however, whether this difference affects clinical outcomes deserves further study.

GM has been shown to play a unique role in the diversity of bile acids in bile acid pools by bile salt hydrolase and bile acid-inducible enzymes (Long et al., 2017; Ramirez-Perez et al., 2017; Mullish et al., 2018). *Eubacterium* and *Ruminococcus* have been proved to contain bile acid conversion enzymes (Jia et al., 2018); we found *Eubacterium* sp. UNK MGS 26, *Ruminococcus bromii*, *Eubacterium ventriosum*, and *Eubacterium eligens* associated with blood bile acids level according to Spearman analysis. These bacteria belong to *Eubacterium* and *Ruminococcus*, so they may also have the ability to convert bile acid.

In conclusion, we confirmed that LT could significantly improve the GM in children with BA and described the characteristics of GM after LT. It provides new insight into understanding the role of LT in BA.

## Data Availability Statement

The datasets presented in this study can be found in online repositories. The names of the repository/repositories and accession number(s) can be found below: NCBI SRA (https://www.ncbi.nlm.nih.gov/bioproject PRJNA730640), it can be accessed with the BioProject identifier PRJNA730640.

## Ethics Statement

The studies involving human participants were reviewed and approved by the Beijing Friendship Hospital, Capital Medical University (Approval ID: 2019-P2–131-02). Written informed consent to participate in this study was provided by the participants’ legal guardian/next of kin.

## Author Contributions

WS: study design, data collection, analysis and interpretation of the data, and writing of the report. L-YS: study design, study supervision, and critical revision of the manuscript for important intellectual content. Z-JZ, LW, WQ, and Z-GZ: clinical treatment assistance. Y-SY: critical revision of the manuscript for important intellectual content. All authors read and approved the final manuscript to be submitted.

## Conflict of Interest

The authors declare that the research was conducted in the absence of any commercial or financial relationships that could be construed as a potential conflict of interest.

## Footnotes

1 http://www.ncbi.nlm.nih.gov

## Abbreviations

BA: biliary atresia
LT: liver transplantation
GM: gut microbiota
SCFAs: producing short-chain fatty acids
TLR: toll-like receptor
KO: KEGG ortholog
KEGG: Kyoto Encyclopedia of Genes and Genomes database
LEfSe: LDA effect size
LDA: Linear discriminant analysis
PCoA: Principal coordinate analysis
ALT: alanine aminotransferase
AST: aspartate aminotransferase
ALP: alkaline phosphatase
GGT: γ-glutaryltransferase
TBA: total bile acids
TBIL: total bilirubin
IL-2: interleukin-2
IL-4: interleukin-4
IL-6: interleukin-6
IL-10: interleukin
TNF α: tumor necrosis factor α
IFN- γ: interferon (IFN)- γ
PELD: Pediatric End-stage Liver Disease.
