# Supplementary material for: Characteristics of Gut Microbiota in Children With Biliary Atresia After Liver Transplantation
Source: Front Physiol. 2021 Jun 29;12:704313. doi: 10.3389/fphys.2021.704313 (PMC8273867; doi:10.3389/fphys.2021.704313)
Supplement: Supplementary file 1 [file Data_Sheet_1.docx]

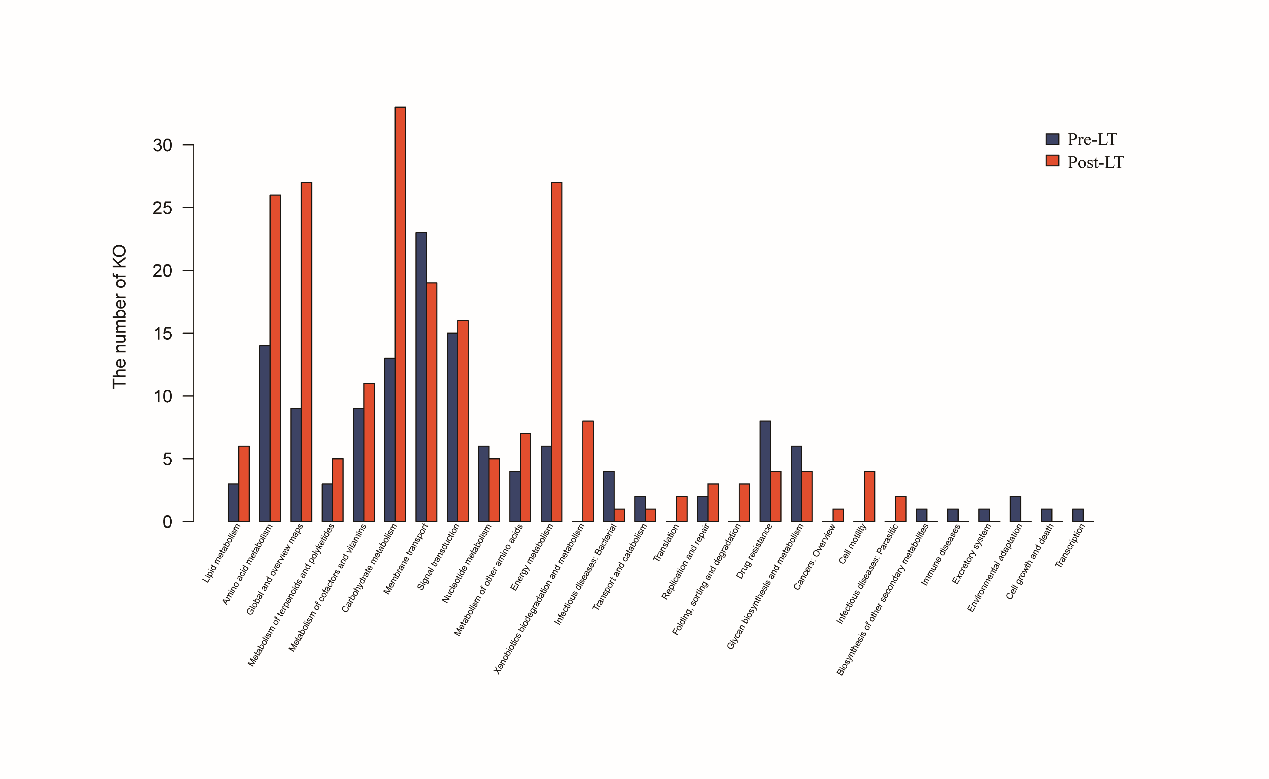
**Fig. S1**

The effect of liver transplantation on the functional pathways at the level 2. The x axis represents the different pathways, and the y axis the number of KO. Different colors represent different groups.


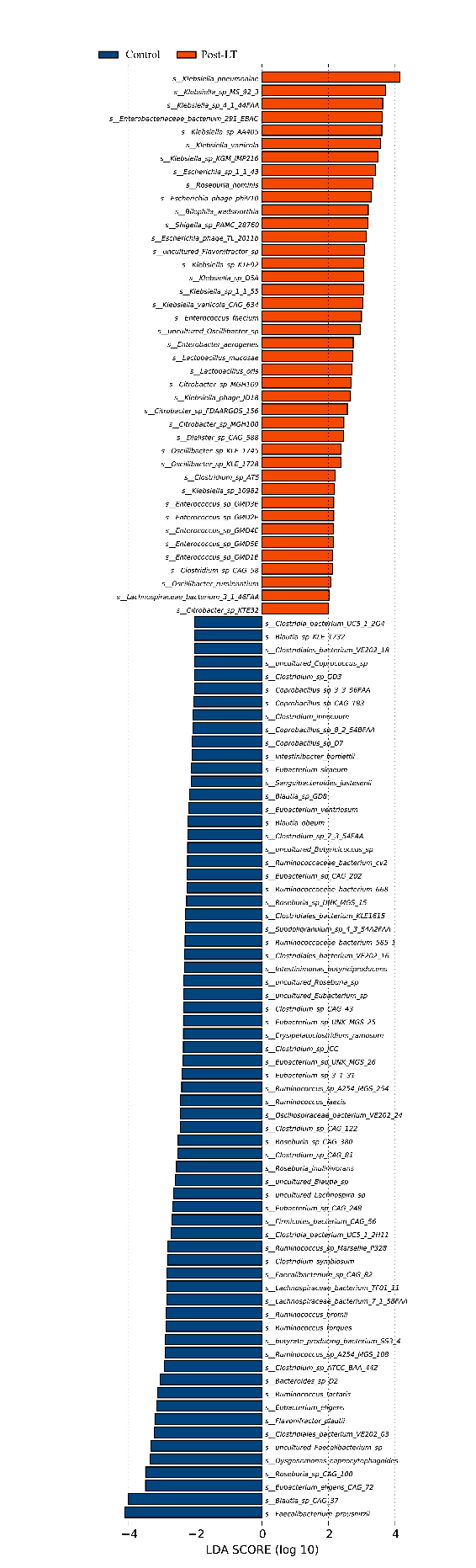


**Fig. S2**

LEfSe analysis of gut microbiota composition between the post-LT and control groups. Histogram of the LDA scores calculated for differential abundance of genera in the two groups. Different colors represent different groups.
